# Same old story with a different ending: Homophily and preferential selection of information within the US climate policy network

**DOI:** 10.1371/journal.pone.0306454

**Published:** 2024-07-01

**Authors:** Lorien Jasny, W. Chris Jayko, Dana R. Fisher

**Affiliations:** 1 Politics, University of Exeter, Exeter, United Kingdom; 2 School of International Service, American University, Washington, DC, United States of America; University of Nigeria, NIGERIA

## Abstract

The passage of the Inflation Reduction Act has been perceived as a substantial shift away from the history of more contentious climate politics in the US. We apply social network methods to interrogate an updated dataset that assesses the degree to which recent policy outcomes are a shift away from earlier policies and positions. We empirically test for homophily, a building block of polarisation, analysing four waves of survey data collected over 12 years from the community of political elites engaged in the issue of climate politics. Using Exponential Random Graph (ERG) modeling, we provide clear evidence that the stances of the top policy actors working on climate change have not shifted substantially. Instead, we document how the policy was successful due to its ability to combine the Administration’s desire to support clean energy along with fossil fuel interests’ aims of expanding extraction and profiting from a transition away from fossil fuels.

## Introduction

With the passage of the Inflation Reduction Act in 2022, the US was celebrated for finally putting the country on the path to meeting its commitments to the Paris Agreement [1–4]. After months of stops-and-starts and years of failed climate legislation, [5–8] the passage of this law has been interpreted as a huge win for the Biden Administration and evidence that the President followed through on his campaign promises to transition the US away from fossil fuels [9, 10]. The passage of what many call “the largest investment the United States has ever made to limit greenhouse gas (GHG) emissions and slow the pace of climate change” [4] also suggests that the political debate about climate change and the necessary transition away from fossil fuels has become less polarised in the US than in previous years [11–13].

This paper applies social network methods to interrogate an updated dataset on the US climate policy network and to assess the degree to which recent policies are, in fact, a shift away from previous policies and positions. It builds on earlier work that documented how ‘echo chambers’ played a critical role in the information transmission among networks of climate policy actors [14–16]. Over one year into implementation of this ground-breaking legislation, this paper investigates what changed to get the bill passed (or the degree to which the climate policy network changed).

Numerous studies have employed a policy network approach to assess the diverse groups of actors who compete to affect policy change [17–19]. This approach has become particularly common among scholars looking at climate-focused networks as a means of understanding the formation of collaboration ties, alliances, advocacy, and power dynamics [8, 20–25]. At the same time, a growing literature has illustrated how increases in political polarisation “greatly affect our capacity to achieve the cooperation that will be necessary to address the challenges facing society over the coming decades.” [26, see also 27, 28]

Polarisation has also been found to affect the function and flow of information within policy networks, which can explain how information flows in the political sphere, and illustrates how these structures can lead to real-world policy outcomes [11, 27, 29]. Specific to climate policy, social scientists have looked to the news media, [30–32] the rise of the conservative countermovement, [33–35] and the polarisation of the issue within the US Congress to explain how a topic with such a high level of scientific consensus can lead to sustained political gridlock [8, 11, 36].

## Data and methods

Our paper takes a policy network approach to interrogate a data set that integrates previous data collected through surveys with the climate policy elite in the US from 2010, 2016, and 2017 with a new wave of data collected in 2022. We constructed the sample of policy elite by comparing organizations and individuals who were identified as central to the US climate policy elite at the time of the specific wave of the study (see SI-1 in S1 File for details on the construction of the sample and SI-2 in S1 File for ethics information, response rates, and a broader description of the data).

Here, we evaluate the six policy-relevant questions that were present in each year of data collection. Our first question, *Human activities are an important driver of current global climate change* (Anthropogenic), references the United Nations Framework Convention on Climate Change’s adoption of the term in 1992 [37]. Over the years, this question has become politically divisive [38]. Respondents’ agreement to this term is considered the most basic level of support for climate policymaking, even while it is debated by sceptics [16], making it a useful barometer. Our second question, *There should be an international binding commitment on all nations to reduce greenhouse gas (GHG) emissions* (Binding), references the history of international agreements which, with the exception of the Kyoto Protocol, have lacked binding mechanisms. Critics of these international bodies argue that without any actual force behind these international resolutions, they are in fact meaningless [10]. Our third question, *The US should meet or exceed reduction target of GHG emissions by 50–52% below 2005 levels by 2030* (GHG), references the commitment made by the United States as part of the Paris Agreement in 2015. The specific rates and years changed in the surveys to reflect the relevant policy discussion at the time of the survey (see S.I-3 for specifics). Asking questions two and three together facilitates the investigation of how respondents might differ on climate policy with respect to international engagement. The last three questions are more geared towards specific policy instruments: *Emissions trading (cap and trade) is the best option for reducing GHG emissions* (CapNTrade). *Federal legislation should include a carbon tax* (CarbonTax), and *The US should provide subsidies for nuclear power as a form of GHG emissions reduction* (Nuclear). Each of these policy mechanisms contain their own pluses and minuses and have been debated within the US. By observing the changing support of these various instruments gives a deeper understanding of the policy space as the same respondents might call the same policy essential, an over-reach, or too little depending on the context. In this paper, we assess if the policy elites in our sample have changed their positions over time, have changed their selection of information sources over time, and whether they show a change in the tendency to prefer information sources more similar to their own position (or what previous research has referred to as an ‘echo’ effect) [14–16]. We achieve these goals through a combination of descriptive statistics and exponential random graph (ERG) models, which are an inferential statistical approach to network data.

In ERG models, tie formation is the dependent variable, and the independent variables are sufficient statistics. These models use simulation methods to test for the presence and significance of heterophily (the preference for information sources with different views), holding other structural tendencies constant [39]. Using these models, we can establish whether organizations are more likely to prefer homogenous sources for their information even when controlling for a variety of other network motifs (For more information on ERG models as well as the control variables used, see SI-5 in S1 File).

## Results

**Fig 1:**
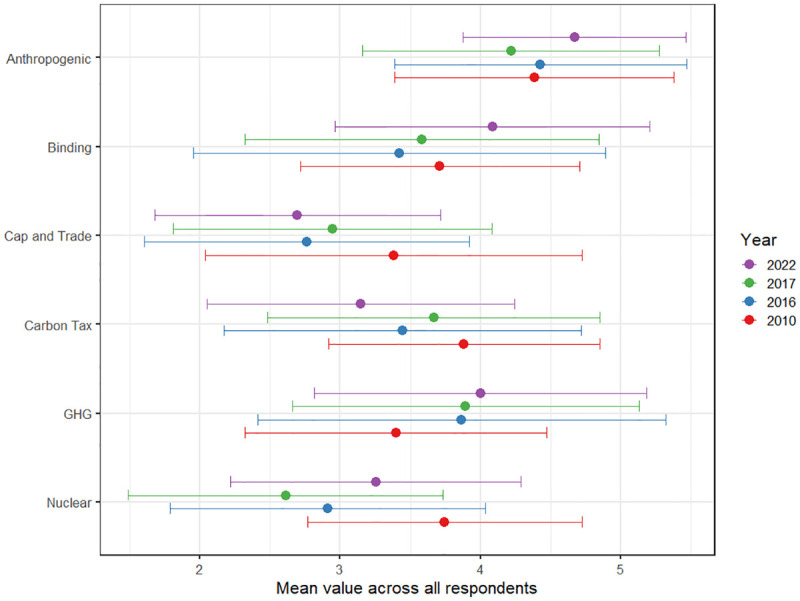
The mean values and standard deviations for all respondents by policy stance over each wave of our sample.

Fig 1 presents the descriptive results of elite responses to each attitudinal question ranging from 1 (strongly disagree) to 5 (strongly agree). The plot shows that there are some small changes in the different variables over time, but the means in the different years are generally similar by question. One exception is the question on greenhouse gas emissions reduction targets, but this question was formulated slightly differently in the 2010 survey–see SI-3 in S1 File for description of the survey questions. We can see, for example, that the mean value for Anthropogenic has changed some (4.38 in 2010, 4.43 in 2016, 4.22 in 2017, and 4.67 in 2022). Like the other survey questions, with the exception of GHG, the changes are not uniformly increasing over time.

**Fig 2:**
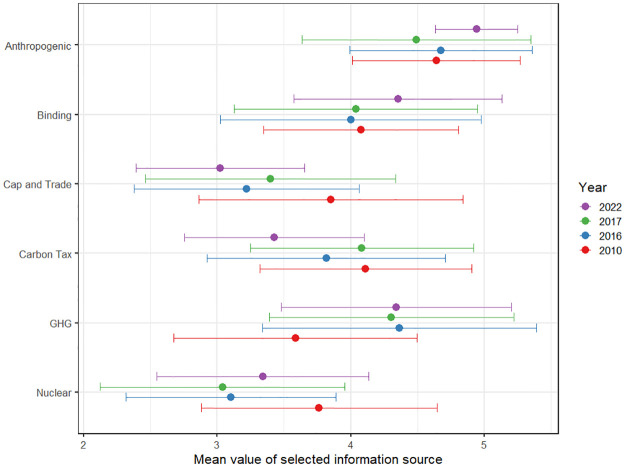
The mean values and standard deviations of the information sources in our networks. This figure includes the same respondent distribution as in Fig 1, but now weighted by how often each elite was cited as a source of expert information on climate change by another respondent.

Next, we look at information transmission within the US Climate Policy Network as measured by the response to our question: *Who are your sources of expert scientific information about climate change*. Respondents were presented with a complete list of our sample of elite political actors and asked to tick off which individuals or organizations from this roster served as their sources (See SI-1 in S1 File for details on the construction of the sample and SI.2 in S1 File for ethics information, response rates, and a broader description of the data. Data are archived on our project site at the OSF: https://osf.io/m3279/?view_only=5f4b0f89cfe04dd183c619470f46079a). This question was administered following the standard methodology for surveying policy networks [40, see also 14–16]. Fig 2 presents the average values per year of information sources in the network weighted by the number of times each actor is a source of information. Once again, we can see that Anthropogenic has the biggest change in mean value over time, where the average information source in 2022 has a value of 4.94 vs 4.64, 4.67, and 4.49 in the other years respectively. Even with this level of variation, though, the differences are not statistically significant. The change in the average source of information mirrors the slight changes in the responses of the population over time, indicating that respondents are not specifically searching out different types of information but changing with the rest of the sample (See SI-4 in S1 File for statistical analyses). To test this relationship in conjunction with other control variables, we turn to Exponential Random Graph Models.

**Fig 3:**
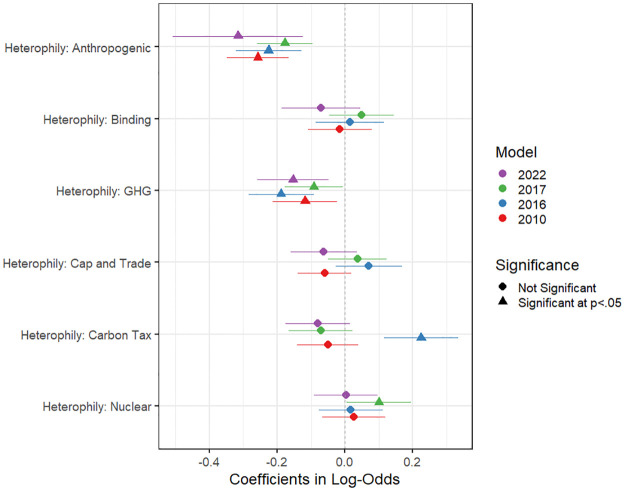
ERG model results for the heterophily terms for each of our policy stances. Full model results are available in the SI-5 in S1 File.

Having documented that there are not notable differences in political elites’ responses to the climate-related policy questions and that their sources of information have not changed substantially over time, Fig 3 presents the results for the heterophily terms, or the difference in opinion, in our ERG models per year (For full discussion of the methods as well as the complete results, which includes numerous control variables, and the goodness of fit statistics for the models, see S1 File). The negative coefficients indicate a narrower difference in opinion, or homophily, which shows the preference for information sources with more similar views. These findings can be interpreted as evidence that, the more a position on a policy issue differs, the less likely an organization or individual is to be selected as an information source by a respondent.

We can see a significant tendency for homophilic preference in both the Anthropogenic and GHG questions in this figure. These effects are consistent across all the time periods, meaning that elite respondents consistently showed a tendency to select information partners who were more similar to themselves on these two items. It is worth noting that we also find statistically significant effects for heterophily for Carbon Tax in 2016 and Nuclear in 2017, which suggests that there are not strong coalitions around these issues [41]. Both these items were not as contentious as Anthropogenic or GHG at the time, but neither were there big changes or fluctuations (as seen in the descriptive results) that might account for significance in these two specific instances. All other terms are not statistically significant, which means that the differences in information source and receiver for these other questions is indistinguishable from simulated networks conditioned on the additional effects included in the model.

**Fig 4:**
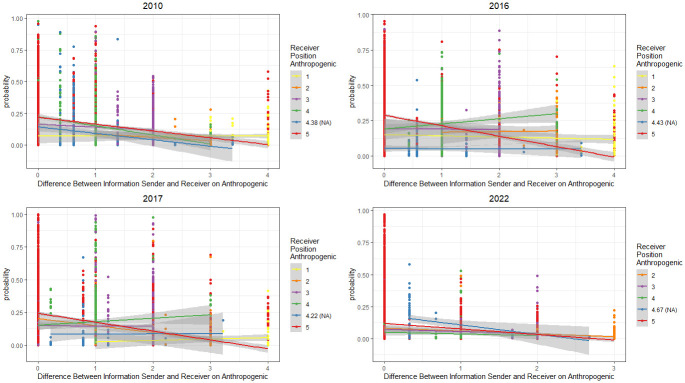
Predicted edge probabilities in each model for the Anthropogenic question. The y-axis shows predicted probabilities for each network tie. Nodes are colored by the receiver’s position on Anthropogenic, and the x-axis measures the difference between the receiver’s position and the sender’s position on Anthropogenic. Downward slopes indicate that, the more different their position are, the less likely it is that a tie will form under this model.

**Fig 5:**
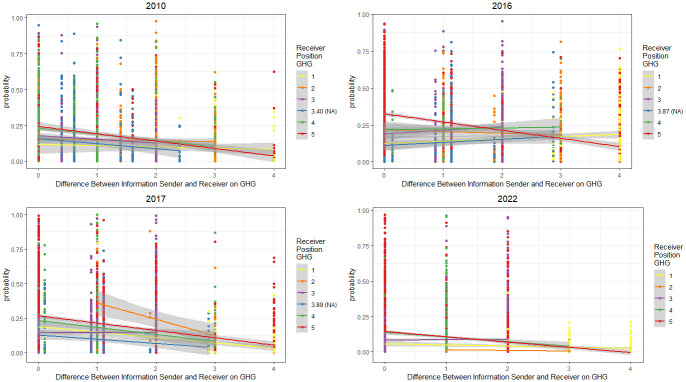
Predicted edge probabilities in each model for the GHG question. The y-axis shows predicted probabilities for each network tie. Nodes are colored by the receiver’s position on Anthropogenic, and the x-axis measures the difference between the receiver’s position and the sender’s position on GHG. Downward slopes indicate that, the more different their position are, the less likely it is that a tie will form under this model.

Finally, Fig 4 looks at the likelihood that a member of the climate policy network will select a source of information by presenting the predicted edge probabilities in each model for the Anthropogenic question (Fig 5 shows the same information for GHG and the remaining results for the policy questions can be found in the appendix). The color of the node indicates the stance of the recipient of the information (the receiver) and the x-axis shows the difference between the stance of the recipient and their source of information (the sender). In 2010, for example, all the lines aside from those at the lowest end of the spectrum (Strongly Disagree on Anthropogenic) are sloped downwards. The downward slope indicates that, as the difference between the information sender and information recipient’s position increases, the probability of the information recipient selecting the sender as information source also decreases. We see this downward slope as a common trend across all years of data for the higher levels of agreement (above neutral). Note that the blue line represents our missing data in each plot. Following previous research, missing data were recoded to the mean value of each variable for each year, but see SI-7 in S1 File, which shows that there are similar results when the missing data is imputed. Interestingly, those at lower levels of agreement (eg Agree–the green line) sometimes slope down (2010, 2022), but also in some years slope up (2016, 2017) showing a slightly greater willingness to engage with others who disagree with them. However, the vast majority of respondents demonstrate a tendency away from engaging with elites with different positions and towards more polarised preferences. See SI-6 in S1 File for the edge probability plots for the remaining variables.

Given the results of these analyses, which clearly document how little has changed within the US climate policy network over the past 12 years, how do we understand the passage of the Inflation Reduction Act (IRA)? As some have noted, in addition to its substantial investments in clean energy, the law is very supportive of the fossil fuel sector [42, 43, for an overview, see also 10, 44]. In addition to surveying the climate policy network, this project also involved conducting open-ended semi-structured interviews with these top policy elites (for details about the methodology, see S1 File). During our interviews in spring 2022 while the policy that became the IRA was being developed and debated, numerous members of the policy network stressed the need for fossil fuel interests to be involved in any successful climate policy. For example, a business association that is focused on energy explained, “we are for all forms of energy that… are available [and] that can be utilized safely and securely.” Many actors, including environmental groups, businesses, and members of Congress mentioned the need for strong support for carbon capture, utilization, and storage (CCUS) as part of any climate policy. At the same time, Democrats in the US Congress also stressed that fossil fuel consumption and production will continue, even with a climate policy in place. As explained by the office of a Democrat who is perceived as a Congressional climate leader: “They’ll still be some of that production happening, but not nearly to the levels that we see right now.” In summary, across all actor types our interviews with policy actors during the 2022 wave of data collection provided additional evidence that their positions regarding a transition away from fossil fuels had not changed. [see also 45] Instead, the policy that was created—the Inflation Reduction Act—integrated their broad interests in expanding clean energy capacity along with supporting fossil fuel extraction and implementation of CCUS.

## Discussion

This study documents how positive political outcomes—like the Inflation Reduction Act—are possible even when polarisation persists. After years of failed efforts to pass climate legislation that would limit carbon emissions by penalizing emitters in the US, a policy was passed that encourages a clean energy transition that does not explicitly harm fossil fuel interests [10, 42]. Instead, the Inflation Reduction Act presents a compromise that incentivizes a transition to clean energy while not punishing fossil fuel interests. By analysing survey and interview data from the top policy elites working on the climate issue that were collected over a 12-year period, we document how this policy emerged even while polarization endured.

In the almost-two years since the IRA was signed, there is evidence that clean energy is being deployed much faster around the US [46]. At the same time, the Biden Administration has continued to support fossil fuel expansion by opening up public lands to development [47]. Based on the most recent data available, while wind and solar power usage have grown, oil, natural gas, and coal continue to be the most consumed energy sources in the US [48]. Moreover, the US became the top exporter of oil and liquified natural gas (LNG) in the world in 2023 [49, 50]. In other words, even as clean energy expands, there is little evidence that the IRA is leading to less fossil fuel extraction. As a result, it is unclear whether this policy that came out of such a polarised political context will achieve its intended climate goals of cutting carbon emissions in the US. A number of recent studies have highlighted the importance of analysing the implementation of policies that aim to address climate change and other environmental problems to understand their successes [51–53]. Future research must build on this line of inquiry and assess the environmental effects of the IRA to evaluate the degree to which this climate compromise achieves its goals.

Research is also needed to compare the effectiveness of policies like the IRA that rely on political ‘carrots’ (ie funding to encourage a clean energy transition) versus those that employ political ‘sticks’ in more detail [54]. Results from such analyses will be extremely valuable in thinking through how to craft effective policies around highly polarised issues and in particularly polarised settings. Additional analysis could look at comparing these policy stances with the others we examined that did not display the same levels of homophily. For example, our results find statistically significant effects for heterophily for carbon tax in 2016 and nuclear energy in 2017. In other words, during these waves of our analysis, policy elites were more likely to draw on a broader range of actors around these specific political issues during these moments. Given that neither issue was particularly policy salient during these years, policy perspectives were less polarized and actors were more likely to draw on a more diverse group for information. It is possible that heterophilic discussion on these less polarised issues aided future collaboration while the issue did not have a high level of policy salience [for more details about policy salience, see 55]. To understand this process in more detail will require more research and data that examine how policy elites develop knowledge about issues that are not being debated. How do they explain the differences between their sources of information for more and less salient policy issues? When policy elites with differing views receive new information, does it stand any chance of affecting issue change (network influence) or is this entirely a store of network selection whereby elites are selecting information sources because they already know their policy stances? In the latter case, changing the information received is likely to have no impact on polarisation at all.

Methodologically, our research also points to opportunities for future research. Our sampling design is intended to track the policy actors most engaged in the climate issue in the recent sessions of Congress (for details, see S1 File). As a result, our sample of elites has changed quite a bit over the past twelve years as the policy actors that are most involved has changed. Consequently, we do not have adequate overlap over to facilitate looking at the dynamics of polarisation longitudinally beyond using the methods we employ in this paper that focuses on analysing and comparing separate cross sections [see 15, 16 for a discussion of the small sample that is surveyed in multiple waves]. Future research could involve data collection efforts that prioritize options for longitudinal analysis so that the social network mechanisms of polarisation (for example, selection vs influence) can be compared in a more comprehensive manner.

In addition to thinking across samples over time, future research should also focus on the differences within the samples. In particular, it should explore what impact there is, if any, for those ties where information sources differ in their policy stances from the respondents (the information receivers). Are the information receivers aware of the differences in opinion, and if so, how does that factor into how they approach the information they receive? Moreover, do we see any significant differences in how the receivers that draw from sources with more and less homogenous perspectives are perceived in the network? Experimental research, possibly in other policy contexts, is needed to investigate whether changing respondents’ sources of information to include more variation would affect the willingness to engage with others who hold these differing views, as well as their perceived influence within the network. As political polarisation grows around the world, understanding how polarised political scenarios can still produce meaningful and effective policy is critical for addressing all sorts of contentious subjects, including the climate crisis.

## Supporting information

S1 File Supplementary information is available in the online version of this paper. (DOCX)
