# Correction: Same old story with a different ending: Homophily and preferential selection of information within the US climate policy network

**DOI:** 10.1371/journal.pone.0311429

**Published:** 2024-09-26

**Authors:** Lorien Jasny, W. Chris Jayko, Dana R. Fisher

The third author, Dana R. Fisher, is incorrectly noted as the corresponding author. The correct corresponding author is Lorien Jasny. Lorien Jasny’s email address is: l.jasny@exeter.ac.uk.

The following information is missing from the funding statement: The authors gratefully acknowledge the financial support provided by the University of Exeter, which facilitated the publication of this research.

The correct funding statement is as follows: This research was supported by the William and Flora Hewlett Foundation (Grant #G-1604-150842, https://hewlett.org) to DRF. DRF and LJ received funding from the John D. and Catherine T. MacArthur Foundation (Grant #G-16-1609-151514-CLS, https://www.macfound.org) and the U.S. National Science Foundation (Grant BCS-0826892, https://www.nsf.gov). DRF and WCJ received support from the Swiss Federal Institute of Aquatic Science and Technology (EAWAG, https://www.eawag.ch/en) and the Oeschger Center for Climate Change Research (OCCR) at the University of Bern (https://www.oeschger.unibe.ch/). The authors gratefully acknowledge the financial support provided by the University of Exeter, which facilitated the publication of this research.
