# Supplementary material for: Same old story with a different ending: Homophily and preferential selection of information within the US climate policy network
Source: PLoS One. 2024 Jul 1;19(7):e0306454. doi: 10.1371/journal.pone.0306454 (PMC11216575; doi:10.1371/journal.pone.0306454)
Supplement: S1 File — (DOCX) [file pone.0306454.s001.docx]

Supplementary Information

*SI-1: Constructing a Data Set and Sampling*

Consistent with previous research studying echo chambers in climate policy networks, sampling for this project began by creating a comprehensive dataset of elite United States climate policy actors engaged in policymaking during the period of the study.^[[1]](#endnote-1)^ A dataset was assembled from three publicly available sources. We began with a list of all the policy actors who participated in climate-related hearings in the United States Congress during the two sessions prior to when data collection began: the 115^th^ session (January 2017-January 2019) and the 116^th^ session (January 2019- January 2021). Consistent with previous studies using this data source,^[[2]](#endnote-2)^ a search for all hearings that discussed climate change was conducted through the Government Printing Office (GPO) FDSys search engine, which archives transcripts from Congressional hearings and makes them available for the public record. Using the search terms “global warming,” “greenhouse gas,” and “climate change,” we identified all of the hearings that discussed these issues during the 115^th^ and 116^th^ Sessions of the US Congress (2017-2021).

The searches yielded 1065 total hearings that fit the search term criteria: 512 hearings from the 115^th^ session of US Congress and 553 from the 116^th^ session of US Congress. The contents of each hearing were then reviewed to confirm that the focus of each hearing was actually the topic of climate change. Of the 1065 hearings that mentioned the search terms, 82 hearings were deemed to have the relevant focus on climate change. From these hearings, 146 testimonies were delivered in 18 separate hearings in the 115^th^ session of Congress, and 516 testimonies delivered in 64 separate hearings in the 116^th^ session of the US Congress. Only formal statements were included in the analysis. Comments made during the question-and-answer portion of the hearings were not analyzed.

Next, we tabulated all non-government actors who were registered in the Senate and House to lobby on climate issues during each respective period through the Open Secrets Lobbying database.^[[3]](#endnote-3)^ We counted any organization that made payments between the years 2017 and 2020.^[[4]](#endnote-4)^ Finally, we cross-referenced this list with a roster of all attendees from the United States who participated in the international climate change negotiations (COP-21) in Paris in December 2015.^[[5]](#endnote-5)^ By drawing from these varied sources, we were able to assemble a dataset that measured sustained engagement in the climate policy network over the years leading up to our period of data collection. Although it would have been ideal to include a list of the speakers who were participating in the on-going 117^th^ session of the US Congress as well, since data collection was taking place in the middle of this particular session, a complete list of hearings and participants was not available.

Finally, actors in this dataset were ranked according to the degree to which they participated in hearings, international negotiations, and on lobbyist registries (if they were non-state actors). Testimonies were weighted such that multiple appearances before Congress indicated greater participation. In some cases, policy actors participated in climate-related Congressional Hearings more than once, but in other cases, the actors participated in the Congress once but also participated in the climate negotiations and/or were registered to lobby on the issue. After reviewing the rankings of actors across the 4-year period, we were unable to identify a defensible threshold for participation in the policy network when we included the 115^th^ session. The emergent thresholds either yielded a sample that was too large (155 actors) or too small (59 actors).

Since the 116^th^ session of the US Congress was the first session to include the Select Committee on the Climate Crisis in the House of Representatives, there were ample climate-related discussions during this period. Therefore, we decided to remove participation in the 115^th^ session of the Congress (in hearings or as a lobbyist) from the sampling criteria, as well as participation in the COP-21 round of the climate negotiations, since the meeting had taken place 7 years prior to our period of data collection. After taking these two steps, a clear threshold was identifiable that was relatively consistent with previous samples of policy actors who had been actively involved in climate-related discussions. Any members of Congress who were no longer serving when we were collecting data were also removed from the sample. Finally, since the US had re-joined the Paris Agreement and the Biden Administration was actively pursuing climate policies, the US State Department and White House’s Office of Energy and Climate Change were added to the sample. Thus, our final sample for this wave of data collection includes 110 actors who were participating actively in this “climate policy arena.”

Policy actors fell into eight *types*: (1) Businesses and Business Associations/Trade Groups; (2) Congress-Democratic members; (3) Congress-Republican members; (4) Environmental Groups; (5) Non-Governmental Organizations (NGOs), which includes professional associations and think tanks; (6) Scientists; (7) Subnational Governmental Representatives; and (8) US Executive Branch (which includes representatives from Government Agencies).

*SI-2 Data Collected*

The data analyzed in this paper were collected between February and April of 2022. We compare these data to the data collected during previous waves of this project in 2010, 2016, and 2017. It is worth noting that the 2010 data collection period was a period when climate policy was working its way through the US Congress (and was eventually unsuccessful), the 2016 data collection period involved debate about President Obama's Clean Power Plan, and the 2017 data collection period took place while the Trump Administration was dismantling components of the Obama Administration’s Climate Action Plan and withdrawing the US from the Paris Agreement.^[[6]](#endnote-6)^ The 2022 wave of data collection took place while the United States Congress was considering the Build Back Better Act, which was passed in a substantially reduced form as the Inflation Reduction Act in August 2022.^[[7]](#endnote-7)^

Data collection was conducted in accordance with University of Maryland policies on Human Subjects research (IRB Protocol #1838270-1). The 110 policy actors identified in our sample were contacted to participate in our study as they represent the core of political elites that have the most influence over the policymaking process. Since the COVID19 pandemic was ongoing, data were collected through virtual meetings. Actors were interviewed and administered a survey on Qualtrics. Survey and interview data are analyzed in this paper. In total, survey data were collected from 70 policy actors, representing a 64% response rate, and interview data were collected from 68 policy actors in our sample, representing a 62% response rate respectively. These response rates are consistent with previous waves of the study.^[[8]](#endnote-8)^

Even though respondents included actors from across the political spectrum and from all types of organizational affiliations, we observed some differences between the respondents and the non-respondents in our study.. Table SI-1 includes the distribution of respondents by organizational type.

| Organizational Type | Surveys  N (%) | Interviews  N (%) |
| --- | --- | --- |
| Businesses/Groups | 20 (56%) | 18 (50%) |
| Congressional-Ds | 5 (56%) | 5 (56%) |
| Congressional-Rs | 8 (53%) | 7(47%) |
| Environmental Groups | 13 (81%) | 13 (81%) |
| NGOs | 13 (87%) | 13 (87%) |
| Scientists | 2 (100%) | 2 (50%) |
| Sub-GOV | 6 (43%) | 7 (50%) |
| US Executive Branch (Admin/Agencies) | 3 (100%) | 3 (100%) |
| Total | **70** | **68** |

**Table SI-1: Survey and Interview Data from 2022**

*SI-3 Survey Instrument and Interview Protocol*

The survey itself was comprised of attitudinal questions and network questions. Attitudinal questions asked participants to indicate on a scale of 1 to 5—where 1 indicated strong disagreement, 5 indicated strong agreement, and 3 indicated neutrality—their positions on statements that were deemed salient during the data collection period. All questions were asked in the same manner with one exception. The question on GHG was phrased inversely in 2010 – participants were asked if the targets were ‘too ambitious’ whereas in the remaining years participants were asked if they supported hitting those targets. The numbers used in this question were also changed in each wave to reflect the current policy targets under discussion.^[[9]](#footnote-1)^ For the network question, each of the 110 policy actors in our sample was listed in alphabetical order by actor type, and each respondent was presented with three iterations of this list. Participants were then asked to indicate, in order: those actors whom they identified as their sources of expert scientific information about climate change, those actors or organizations they collaborate with on a regular basis, and those actors whom they perceived to be most influential in climate politics, in any ideological direction. The survey instrument and a clean dataset are available at this archive at the OSF (<https://osf.io/m3279/?view_only=5f4b0f89cfe04dd183c619470f46079a> ).

All policy actors were also asked to participate in a short interview. Interviews followed an open-ended, semi-structured format and were more conversational than scripted.^[[10]](#endnote-9)^ Interviews followed a pre-determined protocol, which focused on the status of climate and energy policy in the United States. This interview method allowed for flexibility, encouraging the interviewer to pursue follow-up questions and the interviewee to express candid responses. Interviews ranged from twenty to ninety minutes in length and were recorded digitally and transcribed prior to analysis. Transcripts of the interviews were coded by hand into broad themes and then re-coded into what were determined to be relevant sub-themes. This paper includes the results of analysis of the responses to questions regarding the various policy actors’ positions on a transition away from fossil fuels.

*SI-4: Statistical Tests*

We tested for an increase in the mean value of each variable over time using Spearman’s ranked correlation test (Table SI-2). Anthropogenic showed no change, Binding and GHG show a small but statistically significant increase, while Cap and Trade, Carbon Tax, and Nuclear show small but significant overall decreases.

| Term | Correlation | Significance |
| --- | --- | --- |
| Anthropogenic | 0.116 | 0.072 |
| Binding | 0.166 | 0.014 |
| Cap and Trade | -0.195 | 0.003 |
| Carbon Tax | -0.238 | 0.000 |
| GHG | 0.191 | 0.004 |
| Nuclear | -0.175 | 0.008 |

**Table SI-2: changes in the respondents’ answers over time**

In looking at the sources of information, we examine whether respondents go to those individuals with different responses on the policy in question. Respondents’ answers are weighted by how often they are a source of scientific information. We compare this distribution of responses to the unweighted distribution of values given by respondents. This comparison shows that, overall, the distribution of sources mirrors the general changes we observed in the population of respondents.

| Term | 2010 |  | 2016 |  | 2017 |  | 2022 |  |
| --- | --- | --- | --- | --- | --- | --- | --- | --- |
|  | Difference | P-Value | Difference | P-Value | Difference | P-Value | Difference | P-Value |
| Anthropogenic | 0.217 | 0.106 | 0.171 | 0.267 | 0.258 | 0.072 | 0.245 | 0.014 |
| Binding | 0.224 | 0.125 | 0.143 | 0.534 | 0.275 | 0.132 | 0.214 | 0.134 |
| Cap and Trade | 0.251 | 0.185 | 0.08 | 0.652 | 0.245 | 0.127 | 0.051 | 0.671 |
| Carbon Tax | 0.159 | 0.232 | 0.056 | 0.780 | 0.308 | 0.062 | 0.136 | 0.330 |
| GHG | 0.111 | 0.453 | 0.284 | 0.211 | 0.311 | 0.070 | 0.299 | 0.051 |
| Nuclear | -0.115 | 0.394 | 0.023 | 0.893 | 0.087 | 0.591 | -0.085 | 0.530 |

**Table SI-3: comparing the distribution of responses to the distribution weighted by how often a respondent is also a source of information**

*SI-5 Exponential Random Graph Models*

Exponential Random Graph Models (ERGM) were first developed in the 1980s and 1990s with the p* models.^[[11]](#endnote-10)^ In these models, tie formation is the dependent variable and the independent variables are a combination of sufficient statistics that can be a combination of motifs based on attribute data (like our receiver and heterophily terms), purely structural terms (e.g. mutual ties) or a combination of both.^[[12]](#endnote-11)^ The empirical counts of these sufficient statistics in the network are then compared to a population of simulated networks that uses Markov-Chain Monte Carlo (MCMC) methods to establish maximum likelihood estimates for the importance of each sufficient statistic in replicating the empirical values as the average value of the reference distribution of networks.^[[13]](#endnote-12)^ Our sufficient statistics are summarized in Table SI-4.

| Term | Image | Explanation |
| --- | --- | --- |
| Edges | 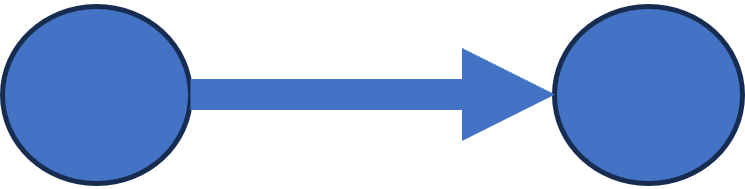 | This term acts like an intercept and represents the base probability of tie formation compared to 50% of ties being present. Negative coefficients indicate that the network is more sparse than 50% density holding all other terms constant. |
| Mutual | 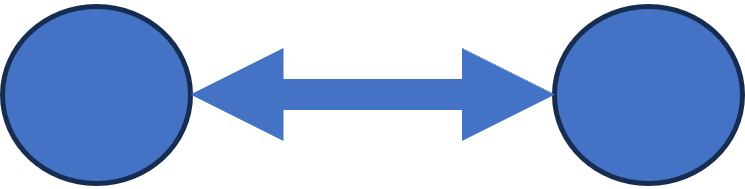 | The tendency for two-way information flow – given that node A selects node B as an information source, how likely is it that B will select A? |
| Gwidegree | 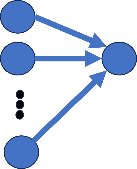 | This term looks at whether some respondents tend to nominate far more sources of information than others. It is reverse-coded, so a negative and significant coefficient here would indicate a tendency for some respondents to have far higher numbers of information sources than others. |
| Gwesp | 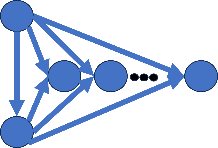 | This term looks at whether nodes that are tied to each other are likely to send information to the same source. This term represents the ‘chamber’ formation used in previous work and clustering in other social networks literature. |
| Gwnsp | 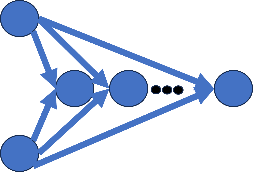 | This term looks at whether joint information sources of a recipient are not tied – it aids in the interpretation of clustering and Gwesp. |
| Receiver – attribute | 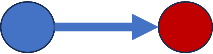 | This attribute term looks at whether respondents at higher levels of our given attributes are likely to seek out more sources of information compared to other values of the same attribute. This term is necessary for correctly interpreting our heterophily term. |
| Heterophily - attribute | 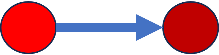 | This is our main term of interest. Here, we look at whether information is more likely to be sent depending on the absolute difference in the value of an attribute for each node. A negative coefficient means that the tie is more likely the more similar the two respondents are on the specific attribute. |

**Table SI-4: Terms used in the Exponential Random Graph Models**

The full model results are presented in Figures SI-1 and SI-2 and Table SI-5. The figures show the control and receiver terms in the same presentation as the results in the main paper and the Table has the numerical version.


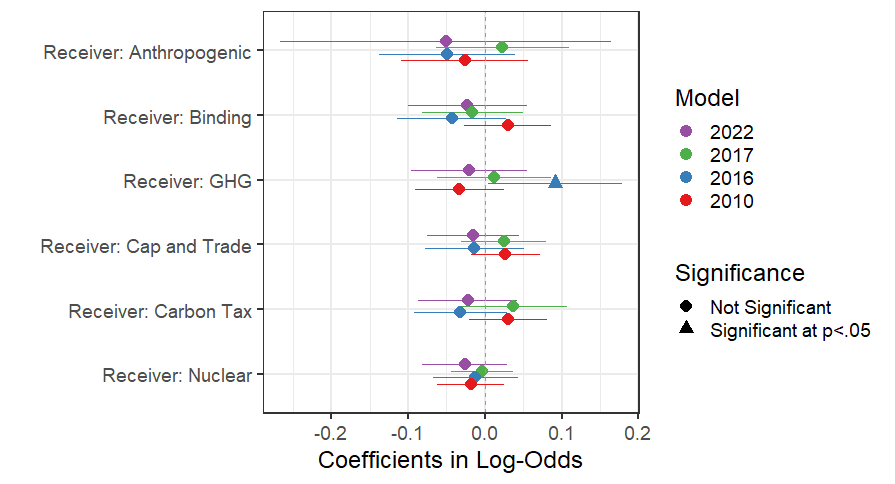


**Figure SI-1: Receiver terms from full model**


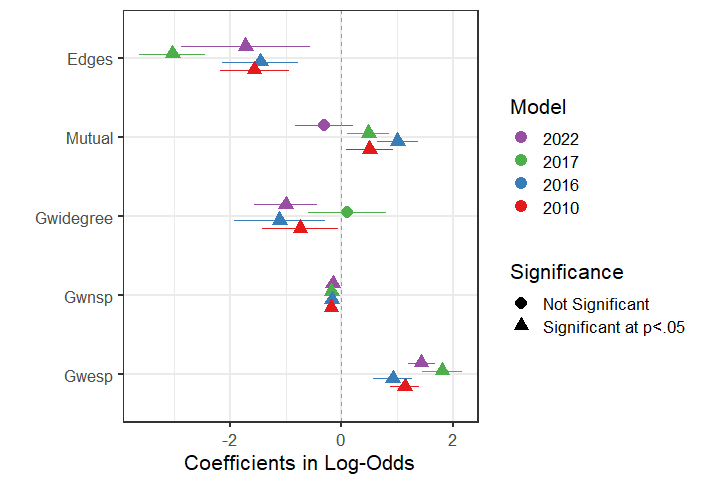


**Figure SI-2: Control terms for the full model**


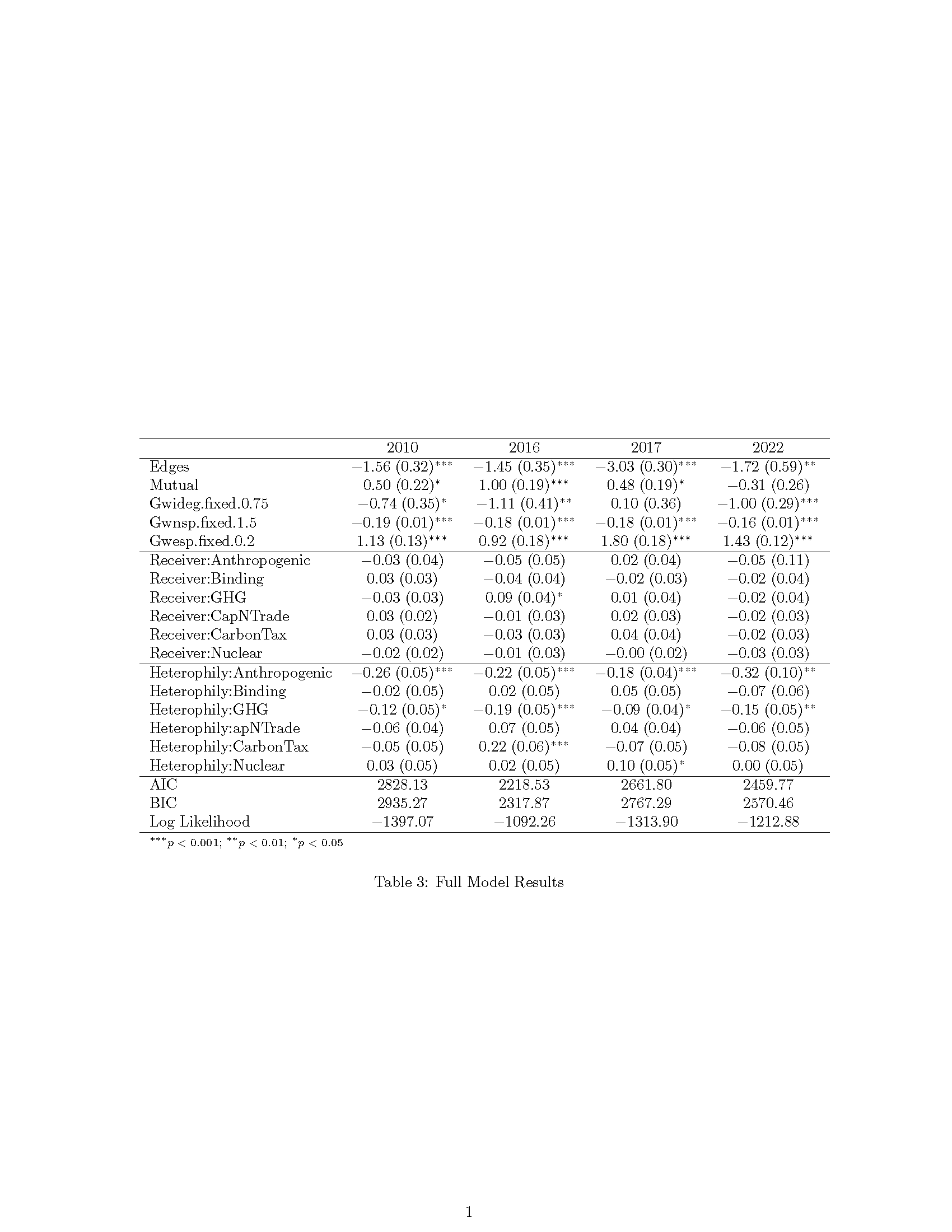


**Table SI-5: Full Model Results**

In addition to the full model results, we also ran a model switching the direction of the sufficient statistics. This next model controls for gwodegree (whether certain respondents are more likely to be named as information sources) and the sender terms (whether higher levels of our attribute variables are more likely to be named as information sources). We would have liked to include these terms in the full model with the indegree terms, but unfortunately this addition caused heavy multicollinearity problems and the models could not converge as multiple terms were too similar. By separating them, we are still able to see that our main conclusion – the importance of the heterophily terms and specifically the importance of homophily along Anthropogenic and GHG – still hold, regardless of whether we model information as incoming (receiver terms as in our main model) or as being sent (this second model).


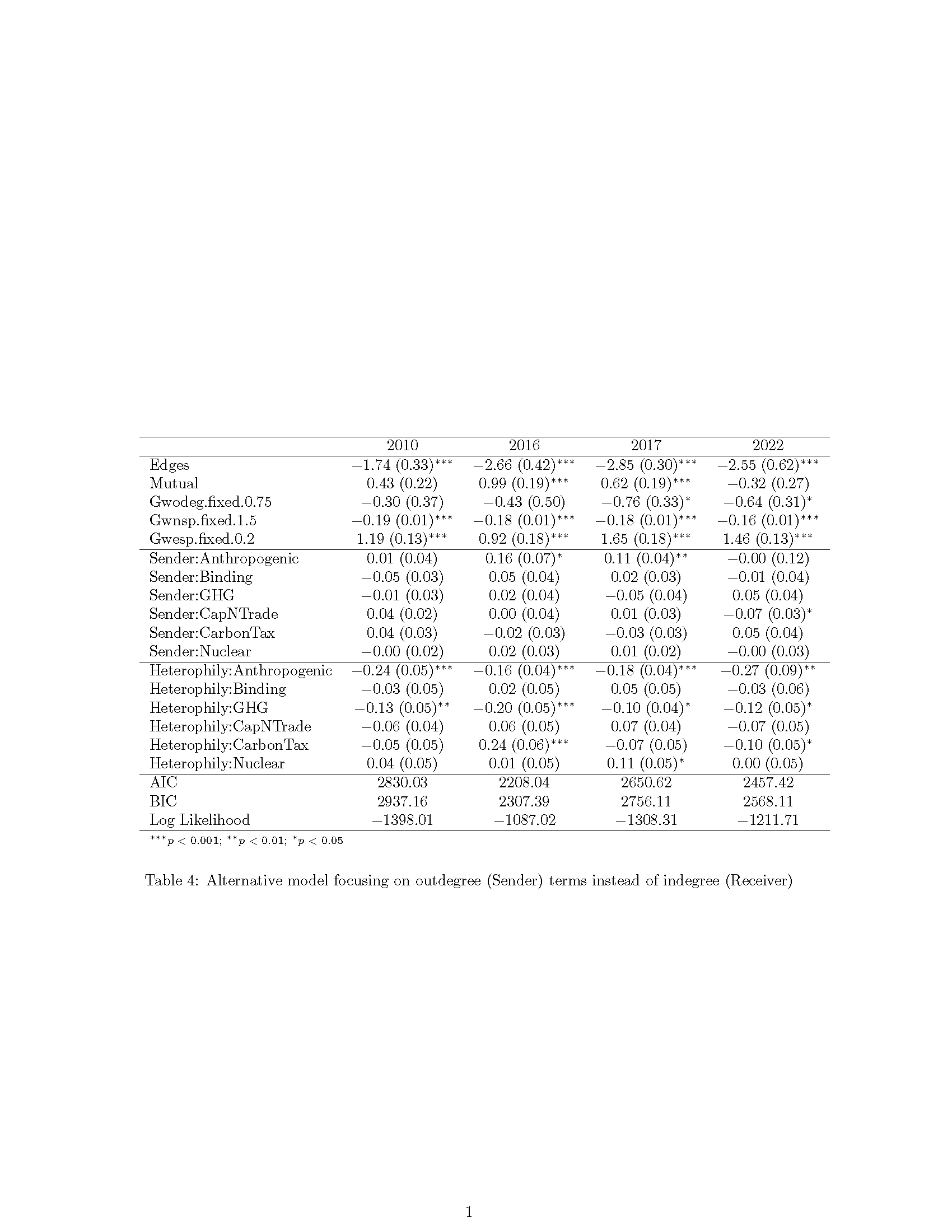


**Table SI-6: Alternative models focusing on the sender of information rather than the receiver**

Finally, Figures SI-3 and SI-4 present the goodness-of-fit results for the full model presented in the paper, as well as the additional model focusing on sent ties instead of received ties. What these images show is that the models converged well and the reference distributions developed by the MCMC simulation. The empirical values are depicted as the black line. They are all relatively close to the mean of the simulations (the bar plots) in the image which show overall goodness of fit of the models.^[[14]](#endnote-13)^


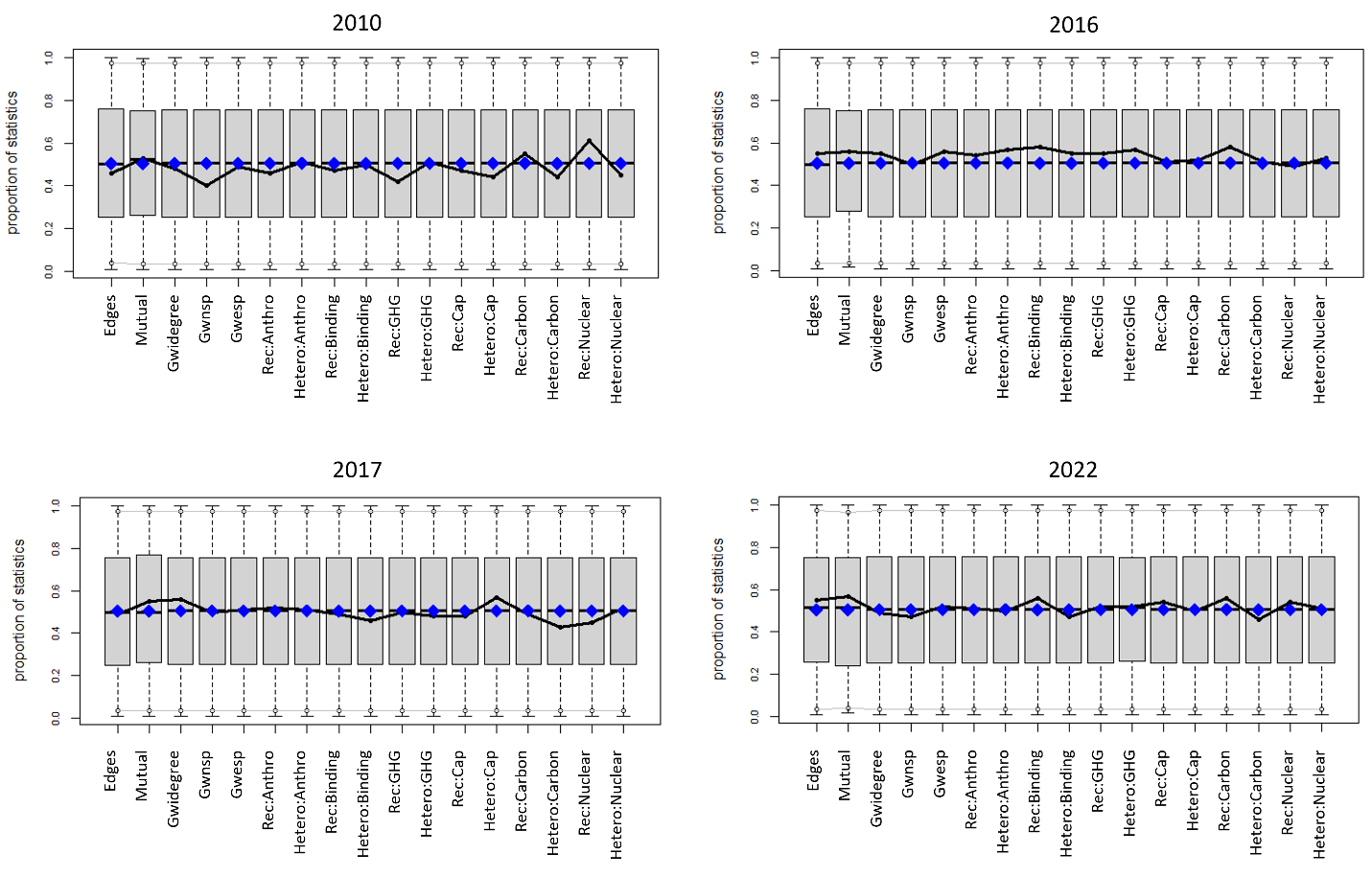


**Figure SI-3: Goodness of Fit for the full model**


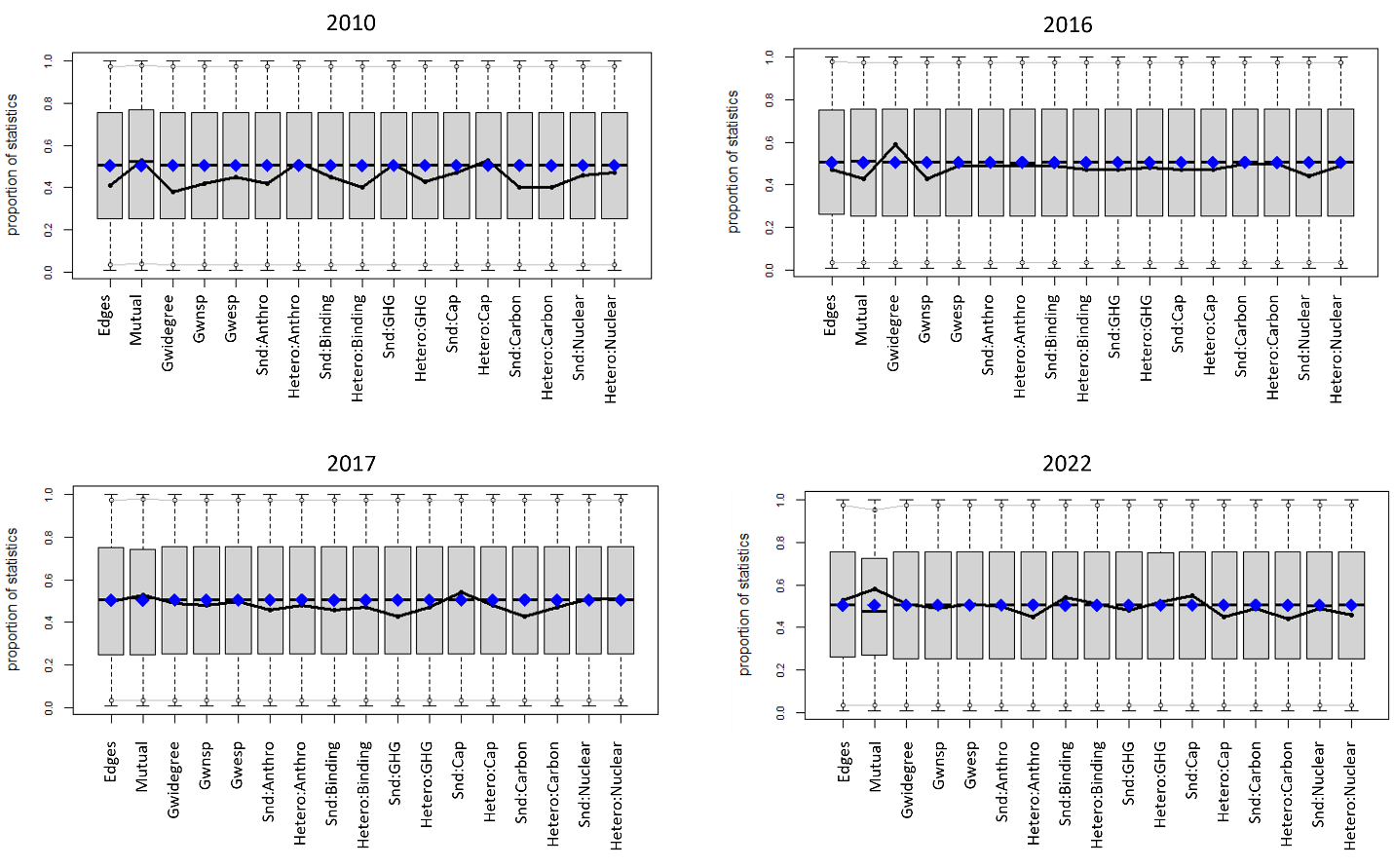


**Figure SI-4: Goodness of Fit for the additional model focused on senders rather than receivers of information**

*SI-6 Additional edge probability images*

Here we show the edge probability predictions based on the other attributes (Anthropogenic and GHG are examined in the full text). Figure SI-5 shows the probabilities for Binding, SI-6 for Cap and Trade, SI-7 for Carbon Tax, and SI-8 for Nuclear. As with the images in the full text, each point represents one edge in the network. The respondent’s (the receiver of information) value of the given attribute is indicated by the colour of the point (see legend), and the difference between the receiver’s value and the sender’s value is given by the x-axis. The corresponding probability of this tie existing is on the y-axis. Thus, a downward slope indicates that as the difference between the receiver and sender increases (in their response on the given attribute variable), the tie is less likely to occur. Although all the plots show similar tendencies towards homophily in the extreme values, the slopes are not as steep as for Anthropogenic nor GHG resulting in the lack of significance we observed.


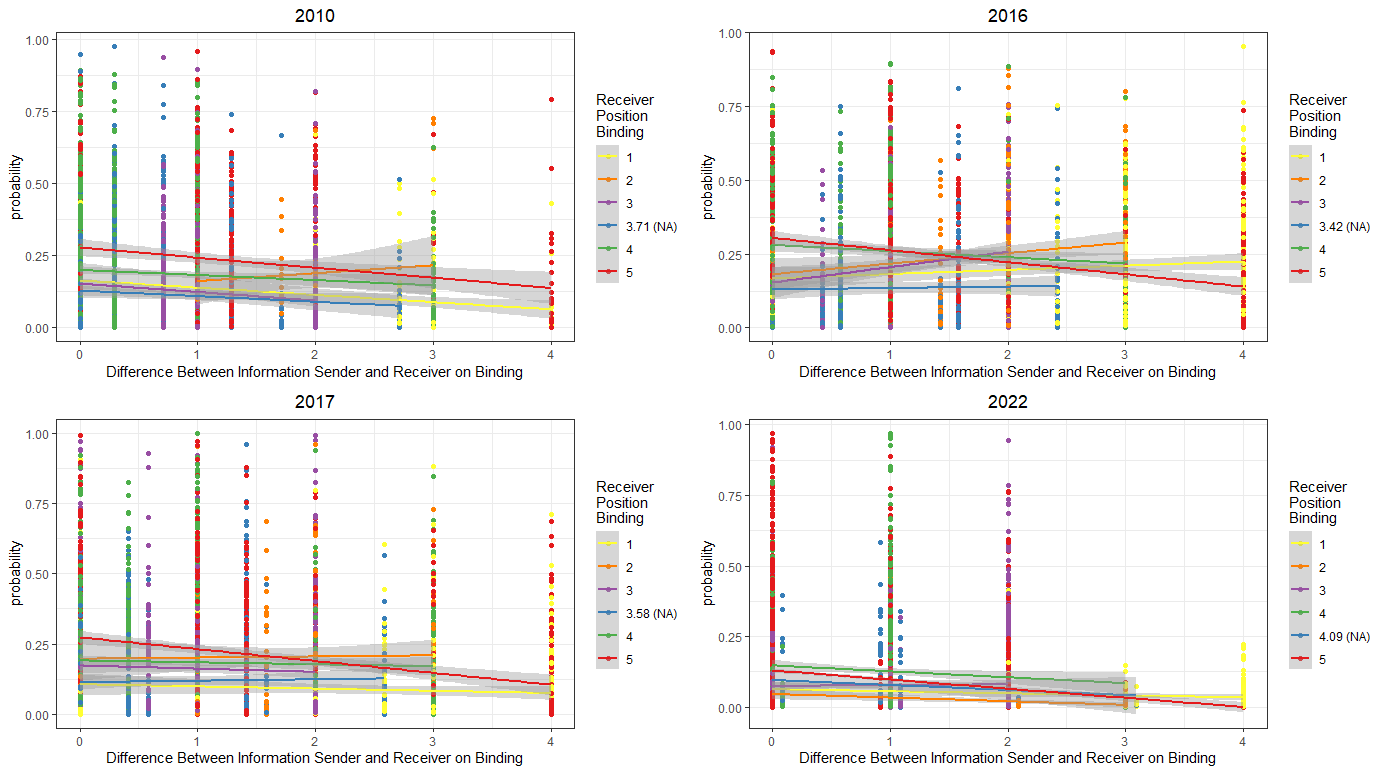


**Figure SI-5: edge probabilities for Binding**


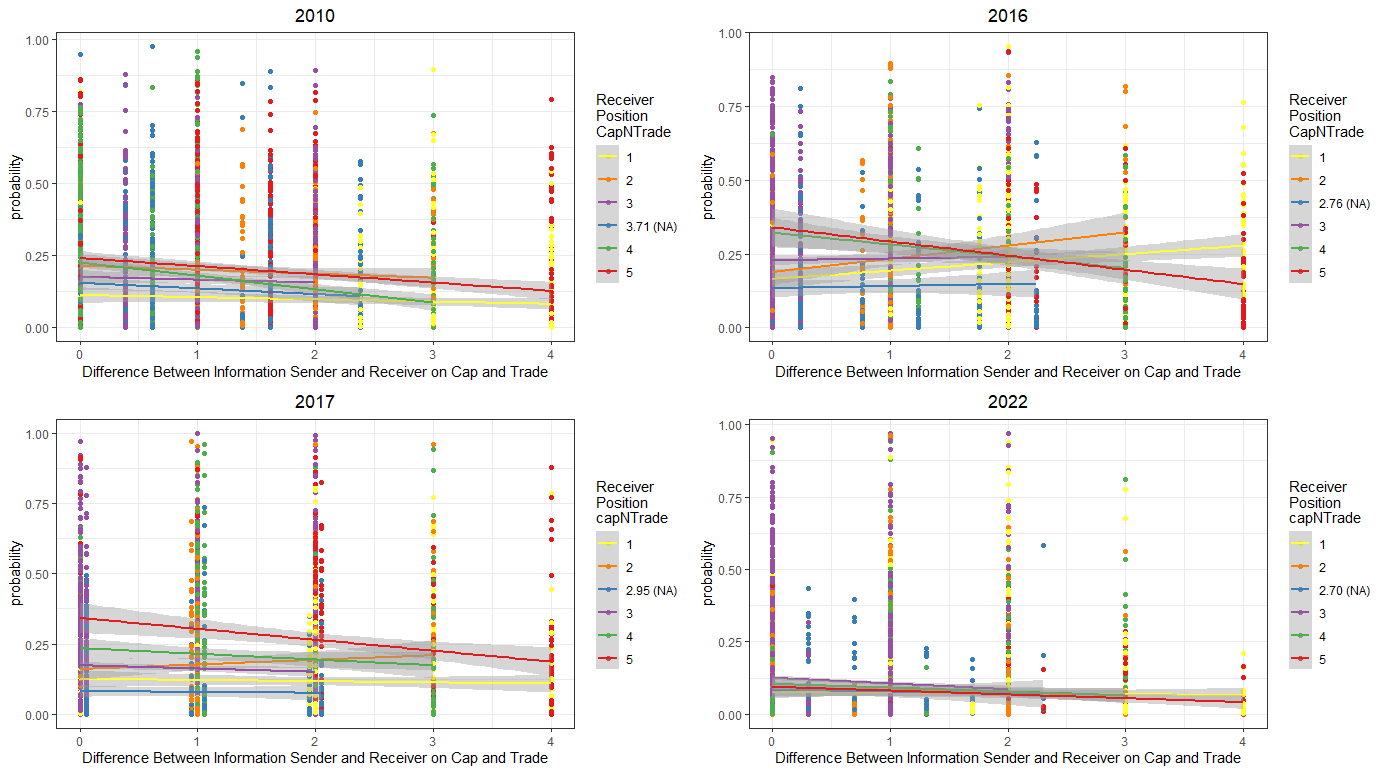


**Figure SI-6: edge probabilities for Cap and Trade**


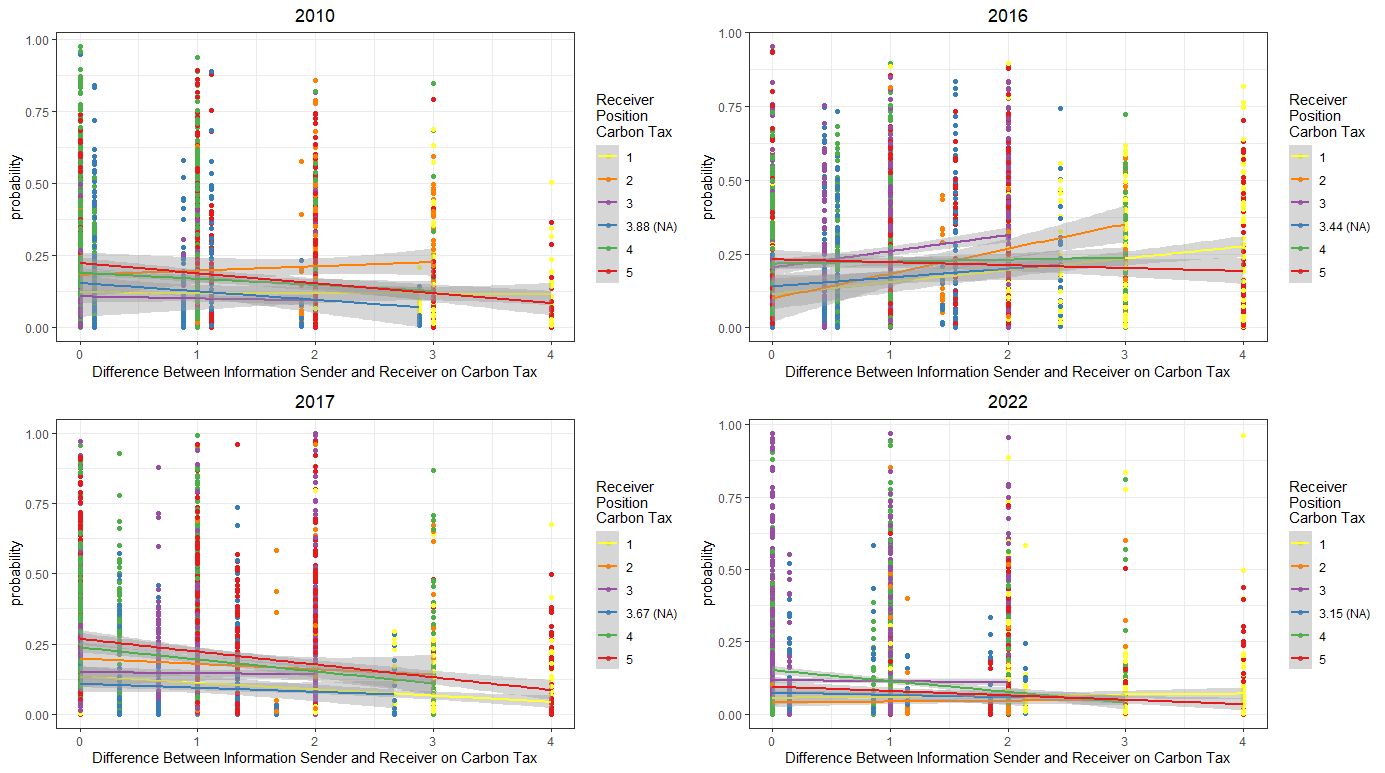


**Figure SI-7: edge probabilities for Carbon Tax**


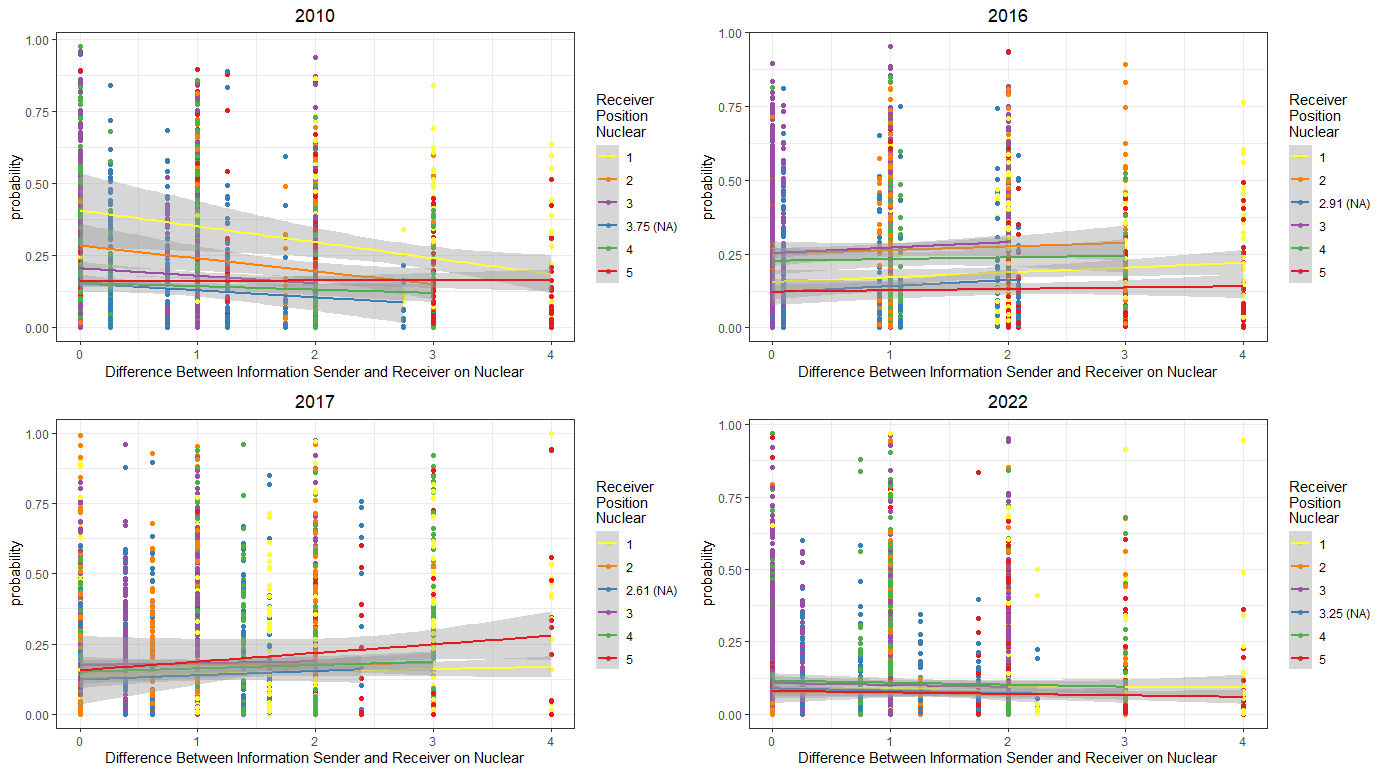


**Figure SI-8: edge probabilities for Nuclear**

*SI-7 Imputed data*

The full text displays results when NA values for the different attributes are replaced by the respective mean values for the different years. We also used imputation methods from the Hmisc package^[[15]](#endnote-14)^ and here display the results from ERG models for 10 different imputed models for each year.^[[16]](#endnote-15)^ We see that the imputed models return almost identical results as the models presented in the main text with the one exception that Heterophily for GHG in 2017 is now not significant, but the remaining years are still significant. Therefore, the imputed data upholds the overall conclusions of the paper.


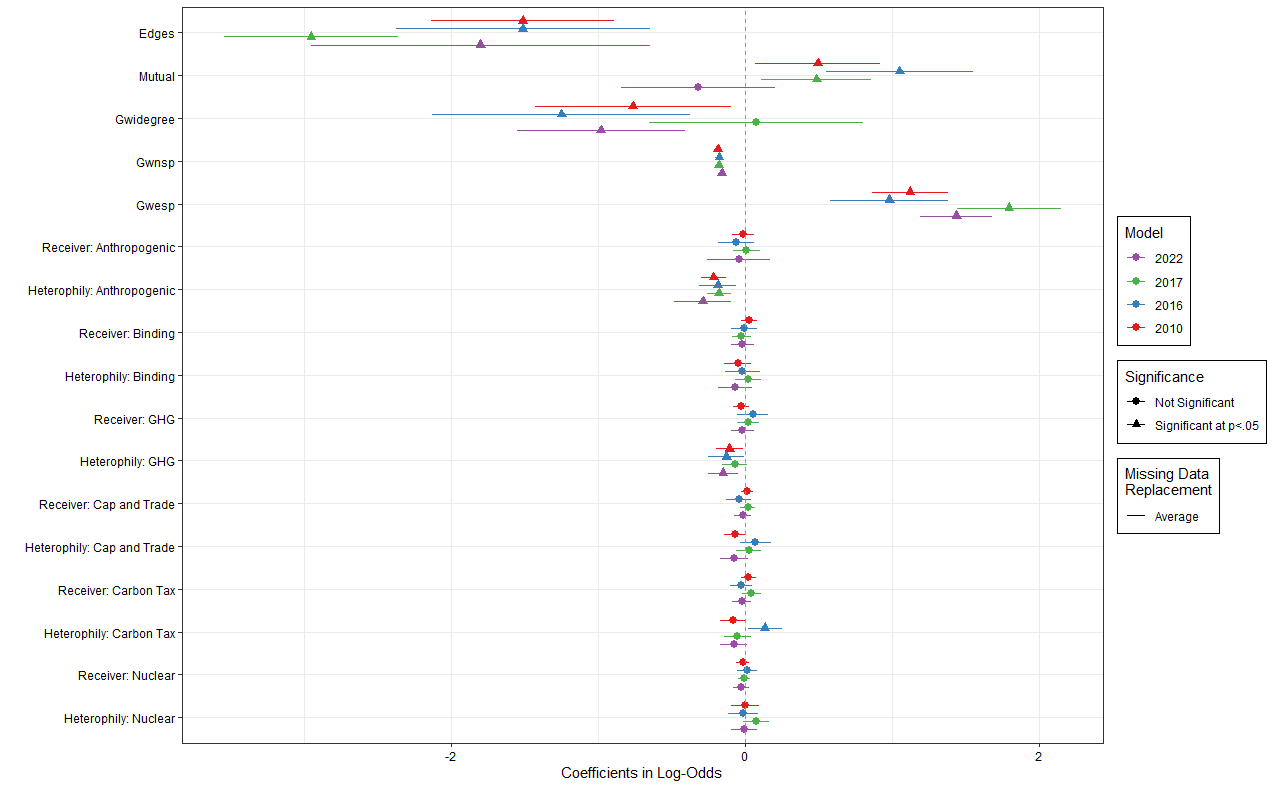


**Figure SI-9: averaged results from 10 ERG models with imputed data**

1. Lorien Jasny, Joseph Waggle, and Dana R. Fisher, “An Empirical Examination of Echo Chambers in US Climate Policy Networks,” *Nature Climate Change* 5, no. 8 (August 2015): 782–86, https://doi.org/10.1038/nclimate2666; Lorien Jasny et al., “Shifting Echo Chambers in US Climate Policy Networks,” *PLOS ONE* 13, no. 9 (September 14, 2018): e0203463, https://doi.org/10.1371/journal.pone.0203463; Lorien Jasny and Dana R Fisher, “Echo Chambers in Climate Science,” *Environmental Research Communications* 1, no. 10 (October 11, 2019): 101003, https://doi.org/10.1088/2515-7620/ab491c. [↑](#endnote-ref-1)
2. Dana R. Fisher, Philip Leifeld, and Yoko Iwaki, “Mapping the Ideological Networks of American Climate Politics,” *Climatic Change* 116, no. 3–4 (2013): 523–45; Dana R. Fisher, Joseph Waggle, and Philip Leifeld, “Where Does Political Polarization Come from? Locating Polarization within the US Climate Change Debate,” *American Behavioral Scientist* 57, no. 1 (2013): 70–92; Dana R. Fisher and Philip Leifeld, “The Polycentricity of Climate Policy Blockage,” *Climatic Change*, July 5, 2019, https://doi.org/10.1007/s10584-019-02481-y. [↑](#endnote-ref-2)
3. <https://www.opensecrets.org/federal-lobbying/> (Accessed 26 October 2021). [↑](#endnote-ref-3)
4. Since the 116^th^ Congress ended in early January of 2021, we did not include 2021 in our sample. [↑](#endnote-ref-4)
5. <http://unfccc.int/resource/docs/2015/cop21/eng/inf03p01.pdf> (Accessed 25 October 2021). [↑](#endnote-ref-5)
6. for a discussion of these political moments, see Jasny, Waggle, and Fisher, “An Empirical Examination of Echo Chambers in US Climate Policy Networks”; Jasny et al., “Shifting Echo Chambers in US Climate Policy Networks”; Jasny and Fisher, “Echo Chambers in Climate Science.” [↑](#endnote-ref-6)
7. Kelly Anne Smith, “Here’s What’s In The Inflation Reduction Act,” Forbes, August 23, 2022, https://www.forbes.com/advisor/personal-finance/inflation-reduction-act/. [↑](#endnote-ref-7)
8. Jasny, Waggle, and Fisher, “An Empirical Examination of Echo Chambers in US Climate Policy Networks”; Jasny et al., “Shifting Echo Chambers in US Climate Policy Networks”; Dana R. Fisher et al., “Polarizing Climate Politics in America,” in *Environment, Politics, and Society*, vol. 25, 0 vols., Research in Political Sociology 25 (Emerald Publishing Limited, 2018), 1–23, https://doi.org/10.1108/S0895-993520180000025001; Jasny and Fisher, “Echo Chambers in Climate Science.” [↑](#endnote-ref-8)
9. The numbers for 2022 are taken from the most-recent 2022 survey and represent the rates of emissions reductions in the current US Nationally Determined Contribution to the Paris Agreement (for details, see <https://www.whitehouse.gov/briefing-room/statements-releases/2021/04/22/fact-sheet-president-biden-sets-2030-greenhouse-gas-pollution-reduction-target-aimed-at-creating-good-paying-union-jobs-and-securing-u-s-leadership-on-clean-energy-technologies/> Accessed 7/16/2023) [↑](#footnote-ref-1)
10. John Lofland and Lyn H. Lofland, “Analyzing Social Settings: A Guide to Qualitative Research and Analysis,” *Belmont, CA: Wadsworth*, 1995. [↑](#endnote-ref-9)
11. Frank, O. and Strauss, D., 1986. Markov graphs. Journal of the American Statistical association, 81(395), pp.832-842; Pattison, P. and Wasserman, S., 1999. Logit models and logistic regressions for social networks: II. Multivariate relations. British journal of mathematical and statistical psychology, 52(2), pp.169-193. [↑](#endnote-ref-10)
12. Lusher, D., Koskinen, J. and Robins, G. eds., 2013. Exponential random graph models for social networks: Theory, methods, and applications. Cambridge University Press: Chapter 7. [↑](#endnote-ref-11)
13. Lusher, D., Koskinen, J. and Robins, G. eds., 2013. Exponential random graph models for social networks: Theory, methods, and applications. Cambridge University Press: Chapter 12. [↑](#endnote-ref-12)
14. Ibid. [↑](#endnote-ref-13)
15. Harrell Jr F (2023). _Hmisc: Harrell Miscellaneous_. R package version 5.1-0,

    <https://CRAN.R-project.org/package=Hmisc> [↑](#endnote-ref-14)
16. This is the identical analysis as presented in the Supplementary Information in Jasny, L., Dewey, A.M., Robertson, A.G., Yagatich, W., Dubin, A.H., Waggle, J.M. and Fisher, D.R., 2018. Shifting echo chambers in US climate policy networks. *PloS one*, *13*(9), p.e0203463. [↑](#endnote-ref-15)
